# MALDI-TOF MS and CD Spectral Analysis for Identification and Structure Prediction of a Purified, Novel, Organic Solvent Stable, Fibrinolytic Metalloprotease from *Bacillus cereus* B80

**DOI:** 10.1155/2015/527015

**Published:** 2015-02-23

**Authors:** Rajshree Saxena, Rajni Singh

**Affiliations:** Amity Institute of Microbial Biotechnology, Amity University, Sector 125, Noida, Uttar Pradesh 201303, India

## Abstract

The ability to predict protein function from structure is becoming increasingly important; hence, elucidation and determination of protein structure become the major steps in proteomics. The present study was undertaken for identification of metalloprotease produced by *Bacillus cereus* B80 and recognition of characteristics that can be industrially exploited. The enzyme was purified in three steps combining precipitation and chromatographic methods resulting in 33.5% recovery with 13.1-fold purification of enzyme which was detected as a single band with a molecular mass of 26 kDa approximately in SDS-PAGE and zymogram. The MALDI-TOF MS showed that the enzyme exhibited 70–93% similarity with zinc metalloproteases from various strains *Bacillus* sp. specifically from *Bacillus cereus* group. The sequence alignment revealed the presence of zinc-binding region VVVHEMCHMV in the most conserved C terminus region. Secondary structure of the enzyme was obtained by CD spectra and I-TASSER. The enzyme kinetics revealed a Michaelis constant (*K*_*m*_) of 0.140 *μ*mol/ml and *V*_max_ of 2.11 *μ*mol/min. The application studies showed that the enzyme was able to hydrolyze various proteins with highest affinity towards casein followed by BSA and gelatin. The enzyme exhibited strong fibrinolytic, collagenolytic, and gelatinolytic properties and stability in various organic solvents.

## 1. Introduction

Proteases are recognized by their catalytic type, that is, aspartic, cysteine, metallo, serine, threonine, and the newly identified asparagine peptide lyases [1]. Metalloproteases are the class of hydrolases that cleave peptide bonds by action of a water molecule which is activated by bivalent metal ions like zinc, cobalt, manganese, or nickel. The water molecule serves as a nucleophile in catalysis and also coordinates with the metal ion as a fourth ligand [2]. The catalytic metal ion is usually coordinated by three conserved amino acid side chain ligands that can be His, Asp, Glu, or Lys amino acid and at least one other residue, which may play an electrophilic role [3].

Metalloproteases exhibit deviant physiological and biochemical properties that account for their therapeutical, pathophysiological, and industrial applications. They are implicated in diseases such as arthritis, cancer, cardiovascular diseases, nephritis, central nervous system disorders, and fibrosis [2]. Enzymes with fibrinolytic and collagenolytic properties have been directly employed in clinical therapy, in the regulation of cellular fibrinolysis, prevention, and cure of thrombotic diseases, as antimicrobial agents for removal of necrotic tissue from burns, wound healing, ulcers, treatment of sciatica and herniated intervertebral discs, isolation of pancreatic islets for transplantation, treatment of Dupuytren's disease, and so forth [4, 5].

The study, elucidation, and determination of protein structure have become increasingly vital in proteomics as the structural configuration of the protein significantly contributes towards its functionality. Identification of any enzyme, its active site, and substrate binding region is paramount in finding its application in medicinal and therapeutic fields. In traditional protein chemistry, proteins were identified by de novo sequencing using automated Edman degradation which is based on successive removal of N-terminal amino acids by chemical methods [6]. However since the last decade, this technique has been ousted by mass spectrometry, which has emerged as the most powerful analytical tool for protein and peptide identification in protein chemistry due to increased sensitivity (femto mole level) and 10-fold increase in speed [7]. Peptide identification using mass spectrometry is based on a simple principle where a peptide is ionized and the peptide bonds are fragmented in an MS-MS spectrometer. Each resulting fragment ion forms a peak in the spectrum at the corresponding mass to charge (*m*/*z*) ratio of the ions which are obtained as peptide masses spectra or the peptide mass fingerprint (PMF) that contain sequence information characteristic of its generating peptide [8]. PMF obtained from MS studies is compared with the theoretical peptide masses of proteins stored in databases by means of mass search programs generating a score for each *m*/*z* comparison or using fragmentation data [8, 9].

In the present work, metalloprotease produced by* Bacillus cereus* B80 was purified and analyzed using MALDI mass spectrometry and its phylogenetic relationship was explored. The secondary structure of the enzyme was studied by CD spectrometry and predicted by I-TASSER. The enzyme was characterized with respect to its various hydrolytic activities and stability to find a potential industry for its exploitation.

## 2. Materials and Methods

### 2.1. Microbial Strain and Enzyme Production

A newly identified* Bacillus cereus* B80 strain (NCBI accession number JQ040533) was selected for the present study. The enzyme was produced in statistically optimized media containing (g/l) sucrose, 5.0; bactopeptone, 50.0; beef extract, 20.0; casein, 20.0; yeast extract, 20.0; and NaCl, 10.0, with 3% inoculum, incubated for 72 h at 180 rpm [10]. The fermented broth was centrifuged at 15,000 rpm for 15 min to remove particulate material. The clear supernatant was used as the crude enzyme.

### 2.2. Protease and Protein Estimation

Protease production was assayed in terms of protease activity observed using casein as substrate [11]. One unit of protease activity was defined as the amount of enzyme required to liberate 1 *μ*g/mL tyrosine in 1 min under the experimental conditions.

Protein was quantified according to Bradford's method [12]. The experiments were carried out in triplicate and standard error was calculated.

### 2.3. Enzyme Purification

Chilled ethanol (95%) was added to the enzyme supernatant at 1 : 2 concentrations and kept at −20°C overnight. The mixture was centrifuged at 15,000 rpm for 20 min at 4°C. The supernatant was subjected to freeze drying for ethanol removal and applied on Sephadex-G75 column (50 × 15 mm; Sigma Aldrich) equilibrated with Tris-HCL buffer (pH 7.5) and eluted in the same buffer at a flow rate 1.0 mL/min. The active fractions eluted from the gel filtration column were pooled and subjected to Q Sepharose column (65 × 10 mm; Sigma Aldrich) preequilibrated with glycine NaOH buffer (pH 9). The fractions were eluted with a linear gradient of 0-1 M NaCl in glycine NaOH buffer at a flow rate of 1.0 mL/min.

For all the purification steps, the sample fractions were assayed for protein content and protease activity using casein as substrate. The enzyme recovery and fold purification were calculated in terms of specific activity.

### 2.4. Effect of Temperature and pH on Enzyme Activity and Stability

The optimum temperature and pH of the enzyme activity were examined by varying the incubation temperature from 30°C to 70°C (at pH 9 for 10 min) and performing the assay with phosphate buffer (pH 6-7) and glycine-NaOH buffer (pH 8–10). To study the thermal stability, the enzyme was incubated at temperatures from 30°C to 70°C for 2 h without the substrate fractions. Enzyme samples were withdrawn at every 30 min and assayed for activity under standard conditions. The stability of the enzyme at different pH values was assessed by incubating the enzyme in different buffers (as above) for 2 h without the substrate fractions and the enzyme activity was assessed at every 30 min under standard conditions.

### 2.5. Electrophoretic Analysis

In order to determine purity and molecular mass of the purified protein, SDS-PAGE was carried out as described by Laemmli [13] using 12% polyacrylamide resolving gel. The gel was silver stained [14] to visualize the protein bands. Furthermore Casein and gelatin zymography were performed according to the modified method described by Garcia-Carreno et al. [15]. Casein [0.2% (w/v) with sodium salt]/gelatin (0.2%) was copolymerized with 12% resolving gel. After electrophoresis, the gel was washed successively with Tris-HCL buffer (pH 7.5) containing 2.5% triton X-100 and glycine-NaOH buffer (pH 9.0). The gel was then incubated in glycine-NaOH buffer overnight at 37°C and stained with coomassie brilliant blue R-250.

### 2.6. Identification of Metalloprotease by MALDI Mass Spectrometry

The purified protein bands obtained in SDS-PAGE were excised, subjected to in-gel trypsin digestion. The resulting digests were analyzed by matrix-assisted laser desorption ionization-time of flight mass spectrometry (MALDI-TOF MS; AB SCIEX TOF/TOF 5800 with LC-MALDI). The *m*/*z* spectra representing the monoisotopic masses of the digested protein fragments were acquired in the MS and MS/MS modes and analyzed by ProteinPilot Software. These peptide mass values were searched against the published databases (NCBInr, SwissProt) using peptide database search engines as MASCOT, Profound, Sequest, Omssa, and PepFrag to obtain information about the identity of the protein. Searches were performed with a minimum mass accuracy of 50 ppm for the parent ions, an error of 0.3 Da for the fragments, one missed cleavage in the peptide masses, and carbamidomethylation of Cys and oxidation of Met as fixed and variable amino acid modifications, respectively. The confidence threshold for protein identification was set to 95%. The obtained protein sequence was aligned with the similar proteins using “ClustalW” (from EMBL-EBI) for determination of homology or similarity with other reported sequences [16]. Conserved domains and active site in the sequence were identified through NCBI and Prosite from exPASy, respectively. A dendogram was constructed to analyze the phylogenetic relation of the newly identified protein.

### 2.7. Circular Dichroism of Metalloprotease

To assess the correct conformation of the new metalloprotease UV circular dichroism (CD) spectrum of the protein was acquired. The far-UV CD spectra in a wavelength range of 190–260 nm were recorded on a circular Dichroism spectrometer with Stop Flow (Applied PhotoPhysics Chirascan; AIRF-JNU, Delhi) in a 1-mm path length cuvette. CD spectra were run with a step-resolution of 1 nm, an integration time of 5 sec, and slit width of 0.6 nm, at 37 uC. The spectra were averaged over two scans and corrected by subtraction of the buffer signal. Data are expressed as the mean residue molar ellipticity (MRE) in deg cm^2^ dmol^−1^ defined as

(1) $$\begin{array}{c} \text{MRE}=\frac{M\theta_{\lambda }}{10dcr}, \end{array}$$

where *M* is the molecular weight of the protein, *θ*_*λ*_ is CD in millidegree, *d* is the path length in cm, *c* is the protein concentration in mg/mL, and *r* is the number of amino acid residues in the protein. The secondary structure contents were calculated by online software K2D2 ( http://k2d2.ogic.ca/) used for analysis of the obtained spectrum to quantify alpha helix, beta sheets, and random coils.

### 2.8. Structure Prediction of Metalloprotease

Structure of the new enzyme was predicted by submitting the deduced protein sequence to the automated I-TASSER service (http://zhanglab.ccmb.med.umich.edu/I-TASSER/) which uses multiple PDB structures depending on its structural conservation, to model different parts of protein. The best model was selected from output based on C-score.

### 2.9. Enzyme Kinetics Studies

The effect of incubation time, enzyme concentration, and substrate concentration on casein hydrolysis was studied. The kinetic parameters of the enzyme were determined by measuring the enzyme activity at different substrate concentrations (0–50 mg). The *K*_*m*_ and *V*_max⁡_ values were determined using Michaelis-Menten equation and the Lineweaver-Burk double-reciprocal graph was plotted with the calculated values.

### 2.10. Substrate Specificity Studies

Various soluble protein substrates (gelatin, casein, azocasein, BSA, keratin, and collagen) at 1% concentration were assayed for their activity with the purified enzyme.

### 2.11. Effect of NaCl on Enzyme Activity at Different pH Values

Aliquots of the enzyme were mixed with NaCl (5 mM and 10 mM) at different pH values (6–10) and the activity was assayed under standard conditions.

### 2.12. Fibrinolytic Assay and Activity

The fibrinolytic activity of the purified enzyme was performed according to the method of Astrup and Müllertz [17]. Fibrin plates of 1 mm thickness containing agarose 1.2%, fibrinogen 0.4%, and thrombin 20 U/mL were prepared. Sample was loaded in the well made on the plate. Distilled water and plasmin were taken as blank and positive control, respectively. The plates were incubated overnight at 37°C and observed.

Quantitative estimation of the fibrinolytic activity was performed by modified method of Datta et al. [18]. Fibrinolytic activity was quantified in comparison to the standard curve plotted using known concentrations of plasmin against its absorbance at 275 nm. One fibrinolytic unit (FU) was defined as the amount of enzyme required to increase the absorbance by 0.01 per min.

### 2.13. Collagenolytic and Gelatinolytic Activity

Collagenolytic/gelatinolytic activity was studied on collagen/gelatin plates of 2 mm thickness containing (%) agarose, 1.0 and collagen/gelatin, 1.0. Sample was loaded in the well bored on the plates and the plates were then incubated at 37°C. After 24 h the plates were flooded with coomassie blue R250 staining solution for visualization of the clear zones.

### 2.14. Stability in Organic Solvents

Purified protease was incubated with 50% (v/v) organic solvents (hexane, acetone, butanol, ethyl benzene, xylene, benzene, chloroform, amyl alcohol, ethanol, acetonitrile, toluene, DMSO, propanol, and glycerol) at 37°C with constant shaking (120 rpm) for 10 days. Samples were removed at every 24 h and the residual activity was estimated. Sample without any solvent served as control and enzyme control activity at day 1 was taken as 100%.

## 3. Results

### 3.1. Enzyme Purification

The enzyme was purified to 1.3-fold showing 100% recovery after ethanol treatment. Further purification of the enzyme with Sephadex gel filtration column resulted in 5.55-fold purification and 49.35% recovery. In the final step of purification with Q Sepharose ion exchange column, the enzyme was purified to 13.1-fold with 33.5% recovery (Table 1).

### 3.2. Effect of Temperature and pH on Enzyme Activity and Stability

**Figure 1:**
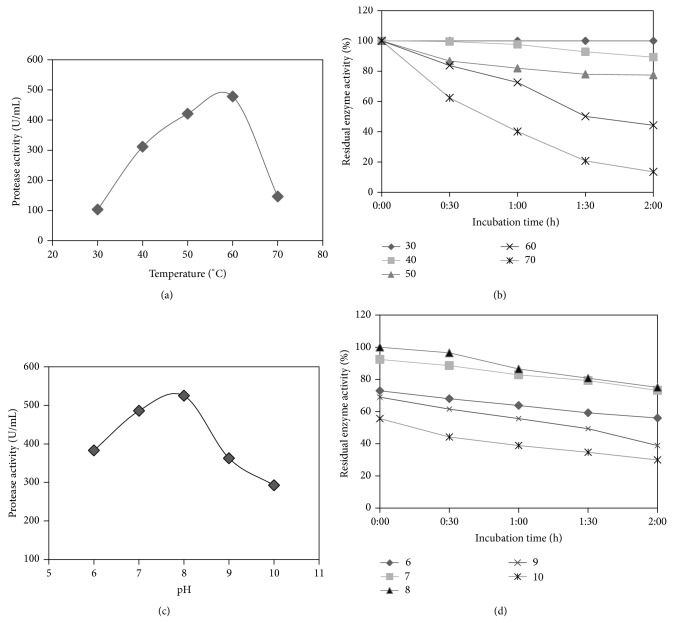
(a) Enzyme activity at different temperatures [30°C to 70°C], (b) stability of enzyme at temperatures from 30°C to 70°C, (c) enzyme activity at different pH values [6 to 10], and (d) stability of enzyme at pH 6–10.

The highest enzyme activity was detected at 60°C (Figure 1(a)), while it was stable for 2 h at 30°C. At 40°C and 50°C, the enzyme lost only 16–22% of its activity after 2 h. At 60°C, the enzyme retained 70 and 44% of its activity after 1 h and 2 h, respectively. At 70°C, the enzyme retained 50% of its activity (Figure 1(b)). The enzyme was active between pH 6.0 and pH 9.0 with maximum activity at pH 8.0 when incubated for 10 min at 60°C (Figure 1(c)). Within this range the enzyme retained about 75–80% of the initial activity, while at pH 10 the enzyme retained about 60% of its initial activity (Figure 1(d)).

### 3.3. SDS-PAGE and Zymography Metalloprotease

**Figure 2:**
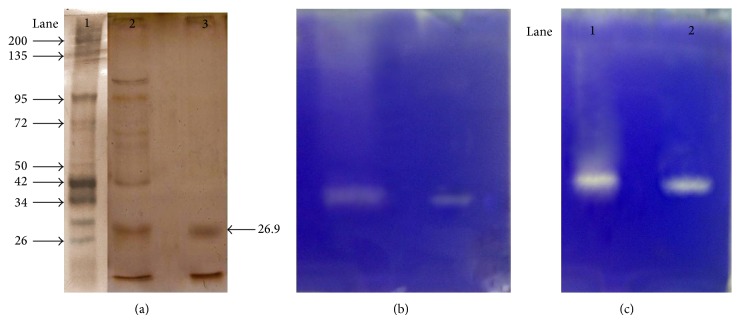
(a) SDS-PAGE of purified metalloprotease. Lane 1: DNA markers of different molecular weights; Lane 2: crude enzyme; Lane 3: purified metalloprotease. (b) Casein zymogram of the purified metalloprotease. Lane 1: Crude enzyme and Lane 2: purified enzyme showing zone of clearance on casein polymerized gel. (c) Gelatin zymogram of the purified metalloprotease. Lane 1: crude enzyme and Lane 2: purified enzyme showing zone of clearance on gelatin polymerized gel.

The purified protein was detected as a single band with a molecular mass of 26 kDa approximately in SDS-PAGE (Figure 2(a)), which corelated with the clear hydrolytic bands observed in casein and gelatin zymogram (Figures 2(b) and 2(c)).

### 3.4. Metalloprotease Identification Using MALDI Mass Spectrometry

**Figure 3:**
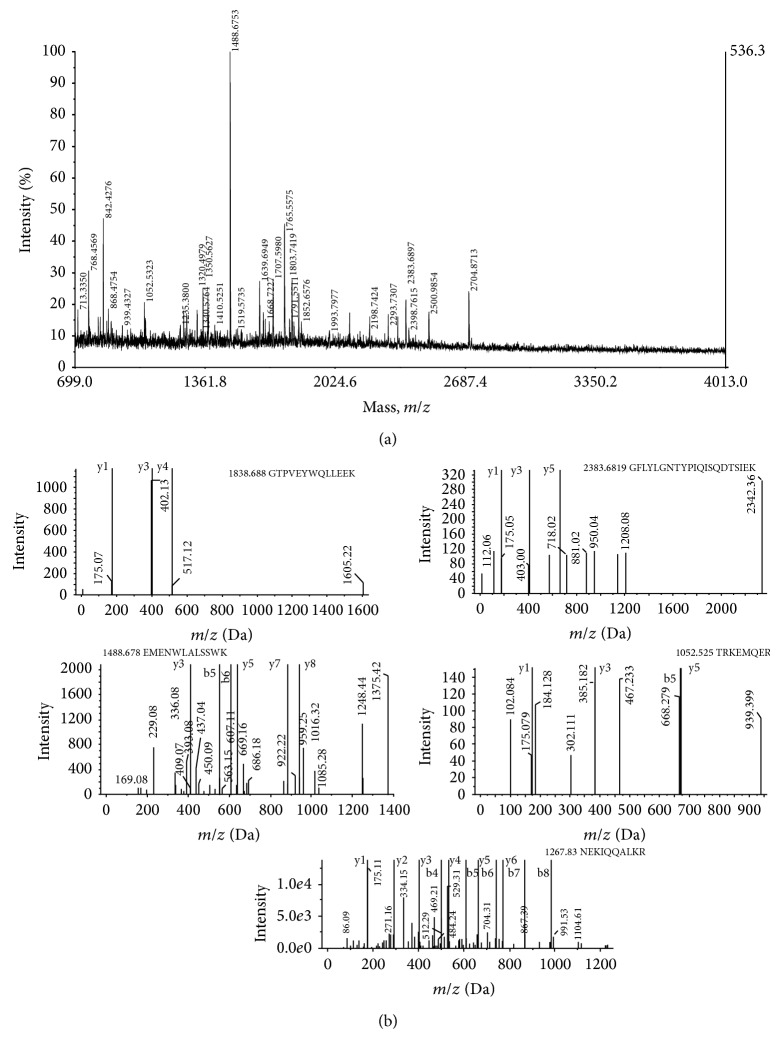
(a) Peptide mass spectra of the tryptic digested peptides as obtained from MALDI TOF mass spectrometry. (b) Annotated MS/MS spectra of fragmentation of 5 peptides in MS/MS that produce mostly y and b ions. The parent *m*/*z* values and the sequence of the identified peptides are indicated.

The MALDI TOF MS analysis of the tryptic digested enzyme generated a spectrum of peaks representing the *m*/*z* ratio of each peptide fragment (Figure 3(a)). Automated analysis of the spectra was performed by software Protein Prospector Auto MS-Fit. Five major peptides were further analyzed on nanospray TOFMS/MS which fragmented the peptides into y and b ions, indicating the best matching peptide sequences (Figure 3(b)).

### 3.5. Database Search

**Figure 4:**
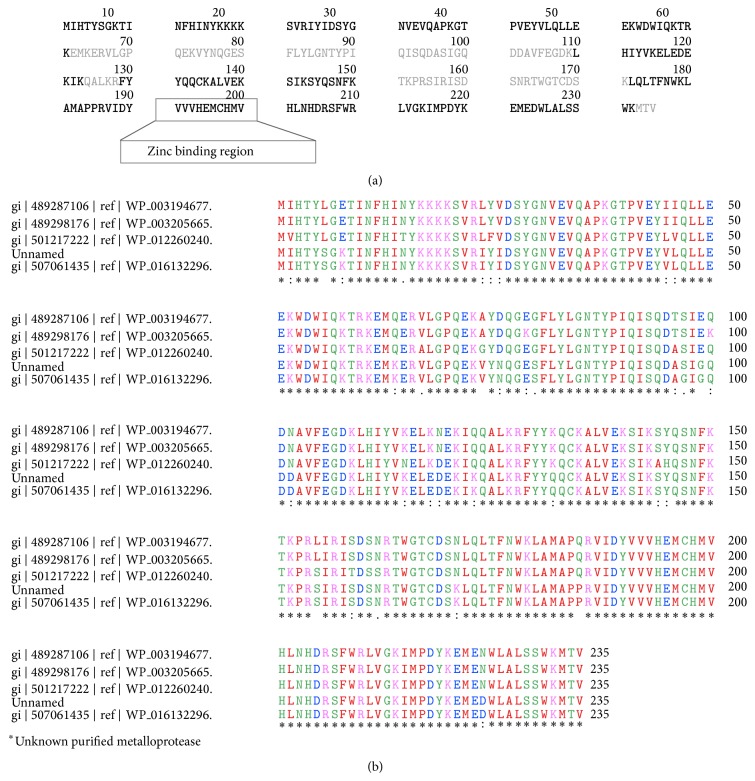
(a) The peptide sequence of zinc metalloprotease from* Bacillus cereus* (gi|507041200) showing matching for *m*/*z* data of the new metalloprotease (in bold). (b) Multiple sequence alignment of the purified protease with similar known sequences using ClustalW.

The PMF searches with MASCOT search engine showed matching of six values (1308.69, 2186.07, 1251.66, 862.38, 830.41, 1324.69, and 1641.8) with a statistically significant score of 94 matching hypothetical protein HPHPM1_1713 [*Helicobacter pylori* Hp M1] (gi|393135873) which demonstrated a score of 24.6 with 22–24% identity with bacillolysin [*Bacillus cereus*; gi|401187907] and M6 family metalloprotease domain-containing protein from various* Bacillus cereus* strains [gi|402425459; gi|507050904; gi|507054527; gi|401287019; gi|401220725]. The database search with Profound, however, yielded more significant results exhibiting 1.0 expectation value with zinc metalloprotease from* Bacillus cereus*; gi|507041200 and 0.79 expectation value with* zinc metalloprotease from Bacillus mycoides *Rock3-17 [gi|228995693] and* Bacillus mycoides* Rock1-4 (gi|229003322) with some unmatched peptides though. The matching of the *m*/*z* data with zinc metalloprotease from* Bacillus cereus*; gi|507041200 is stated in Table 2 and Figure 4(a). The unmatched peptide masses were further searched in Mascot and Profound. The motif search with Prosite revealed the presence of zinc-binding region VVVHEMCHMV in the most conserved C terminus region (Figure 4(a)).

### 3.6. Sequence Homology, Alignment, and Phylogenetic Studies

The sequence homology search performed through NCBI protein blast showed that the purified protein held 90–93% similarity with zinc metalloproteases from various* Bacillus* strains that belong to* Bacillus cereus* group. Thus, the protein was identified as zinc metalloprotease. The protein sequence was found to have three conserved domains DUF45, COG1451, and WLM. DUF45 family includes amino acids 190–240, with C terminus being the most conserved region containing three histidines similar to that found in zinc proteases suggesting that this family may also be proteases. COG1451 includes predicted metal-dependent hydrolase and WLM [pfam 08325] is a predicted metallopeptidase domain called WLM (Wss1p-like metalloproteases). The multiple sequence alignment was performed with known sequences of zinc metalloproteases from various strains belonging to the* Bacillus cereus* group, zinc metalloprotease from* Bacillus cereus* [gi|507061435],* Bacillus mycoides* [gi|489287106],* Bacillus mycoides* [gi|489298176], and* Bacillus weihenstephanensis* [gi|501217222]. The alignment demonstrated the similarity of the purified protease with these sequences (Figure 4(b)).

**Figure 5:**
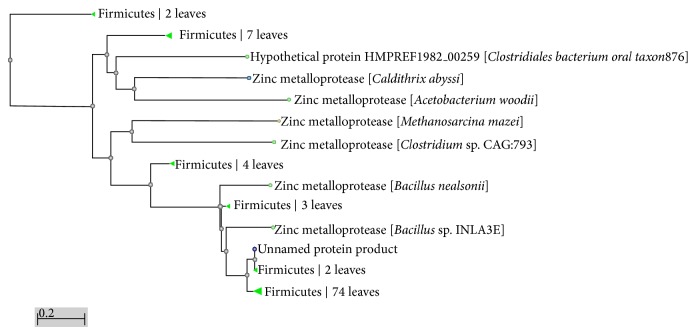
Phylogenetic tree of metallprotease from* Bacillus cereus* B80 with other metalloproteases.

The evolutionary relationship tree (Figure 5) of the new metalloprotease from* Bacillus cereus* B80 showed homology with zinc metalloprotease from various* Bacillus* sp.

### 3.7. Secondary Structure by CD Spectrometry

**Figure 6:**
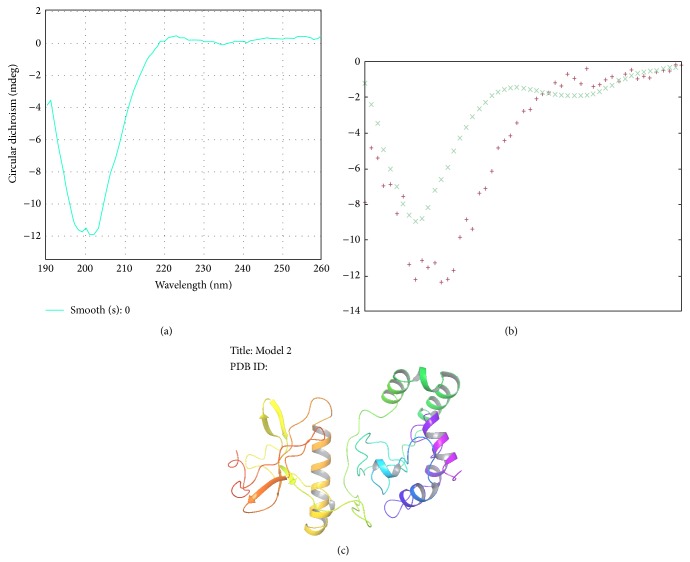
(a) CD spectra of metalloprotease. (b) K2d2 analysis: (+) input spectrum and (×): predicted spectrum of CD spectra. (c) 3D structure (from I-TASSER) of newly identified zinc metalloprotease from* Bacillus cereus* B80.

The CD spectrum of the metalloprotease is represented in Figure 6(a). The spectrum analysis by K2D2 (Figure 6(b)) showed presence of *α* helix, 36.26% and *β* strand, 11.68% with max error more than 0.32. The analysis showed presence of more *α* helices than *β* strands.

### 3.8. Elucidation of Protein Structure

Five predicted models for the newly sequenced zinc metalloprotease from* Bacillus cereus* B80 were obtained from I-TASSER (http://zhanglab.ccmb.med.umich.edu/I-TASSER/) [19]. Model 2 with the highest C-score was selected (Figure 6(c)). The estimated accuracy of Model 2 is as follows: C score: −3.26, TM-score: 0.26 + 0.08, and RMSD: 16.5 + 3.0 Å. C-score is a confidence score for estimating the quality of predicted models by I-TASSER, typically in the range from −5 to 2, where higher value signifies a model with high confidence. TM-score is a recently proposed scale for measuring the structural similarity between two structures. A TM-score >0.5 indicates a model of correct topology and a TM-score <0.17 means a random similarity [20]. RMSD is an average distance of all residue pairs in two structures.

### 3.9. Enzyme Kinetics Studies

**Figure 7:**
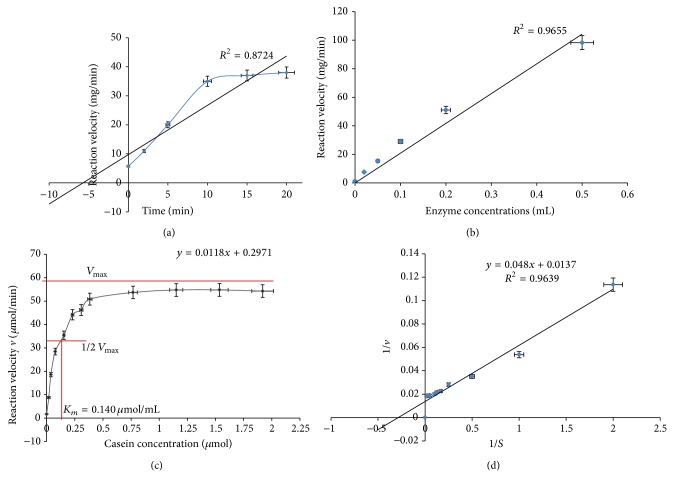
(a) Effect of incubation time on Casein hydrolysis. (b) Effect of enzyme concentration on Casein hydrolysis. (c) Enzyme kinetics, Michaelis-Menten Menton graph and (d) Lineweaver-Burk Plot.

The rate of reaction for the enzymatic hydrolysis studied by varying incubation time is represented by a hyperbola, where the rate of reaction increased from 0 to 10 min, and thereafter velocity of the reaction remained constant. The *R* ^2^ value of 0.872 validates the study (Figure 7(a)).

The enzyme assay with different aliquots of enzyme (0.02 mL–0.5 mL) is depicted in Figure 7(b). The straight line shows that the reaction velocity increases with the increase in enzyme concentration. *R* ^2^ value of 0.9655 validates the study.

The Michaelis-Menten graph was plotted with reaction velocity (*v*) as a function of substrate concentration (*S*) (Figure 7(c)). The kinetic constants values of the purified metalloprotease as obtained from the Michaelis-Menten equation were Michaelis constant (*K*_*m*_) 0.140 *μ*mol/ml and V_max⁡_ 2.11 *μ*mol/min. The Lineweaver-Burk double-reciprocal plot was prepared with the reciprocal of reaction velocity (1/*v*) as a function of reciprocal of substrate concentration (1/*S*) (Figure 7(d)).

### 3.10. Substrate Specificity Studies

**Figure 8:**
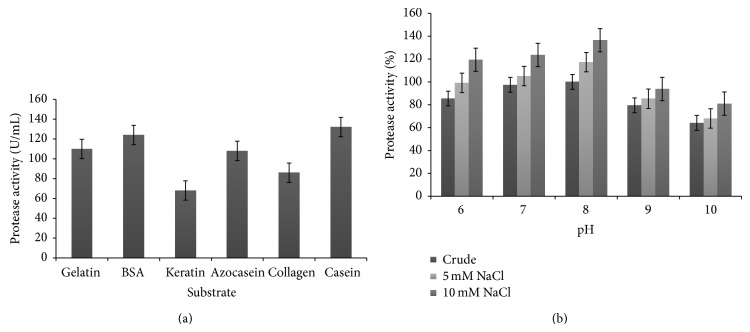
(a) Enzyme activity with different substrates. (b) Protease activity in presence of NaCl at different pH values.

The enzyme was able to hydrolyze all the soluble proteins as gelatin, casein, azocasein, BSA, keratin hydrolyzed, and collagen, with highest affinity towards casein followed by BSA and gelatin (Figure 8(a)).

### 3.11. Effect of NaCl on Enzyme Activity

The enzyme activity enhanced in presence of NaCl at 5 and 10 mM concentrations (Figure 8(b)). This shows that the enzyme is a mild halotolerant protease. It was also observed that NaCl had no effect on optimum pH of the enzyme activity as the increase of about 20% was similar at all pH values (6–9) with maximum activity at 7.

### 3.12. Fibrinolytic Activity

**Figure 9:**
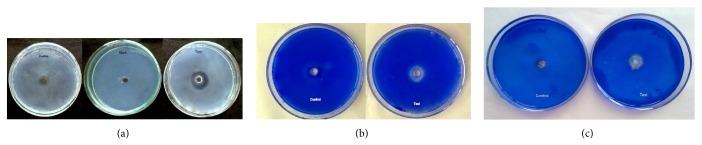
(a) Fibrinolytic activity of the purified metalloprotease against control and positive plates, visible as a clear zone around the well. (b) Collagenolytic activity of purified metalloprotease on collagen plate. (c) Gelatinolytic activity of purified metalloprotease on gelatin plate.

The fibrin plates with the positive (plasmin) and the purified enzyme showed a clear transparent region where fibrin was hydrolyzed, after overnight incubation, indicating fibrinolytic activity of the enzyme (Figure 9(a)). The fibrin plate with the metalloprotease exhibited a bigger zone of hydrolysis than the plasmin plate. Control showed no zone of hydrolysis. The quantitative estimation of the fibrinolytic activity of the purified metalloprotease showed 81FU.

### 3.13. Collagenolytic and Gelatinolytic Activity

The collagen and gelatin plates exhibited a clear zone of hydrolysis on addition of dye after overnight incubation, while the control plates showed no zone of hydrolysis (Figures 9(b) and 9(c)).

### 3.14. Stability in Organic Solvents

The enzyme was highly stable in most of the solvents for 4 days retaining 100–74% activity regardless of their log *P* values (Table 3). The residual activity of enzyme in presence of xylene was 118%, toluene 115%, benzene 83%, ethyl benzene 81%, and amyl alcohol 78% after 10 days. With acetone, the enzyme was stable with 91% activity till day 8, while in presence of DMSO, the enzyme retained 80% activity till the 6th day. The enzyme was less stable with glycerol, chloroform, acetonitrile, ethanol, and propanol retaining only 21, 18, 35, 38, and 28% activity after 10 days and was least stable with butanol retaining only 20% activity after 10 days.

The results also showed that the enzyme activity increased from 100 to 172% in presence of ethyl benzene, 147% with glycerol, 115, 107 and 106% with toluene, chloroform, xylene and DMSO, respectively, on day 1. The activity increased further to 182, 189, 118, 180, 154, 190, 100, 110, and 159% with xylene, toluene, DMSO, amyl alcohol, butanol, acetonitrile, ethanol, acetone, and propanol-2, respectively.

## 4. Discussion

The three-step purification process of enzyme was followed keeping in view the physicochemical nature of the enzyme and other components present in the media after fermentation. The supernatant obtained after centrifugation appeared to be dense and viscous, which might have been due to the presence of DNA and exopolysaccharides. Protein supernatants are treated with ethanol and isopropanol for reduction in viscosity [21]. Proteins are complex ampholytes that have both positive and negative charges depending upon the proportions of ionizable amino acid residues in its structure. However, in metallo-proteins, an internal metal ion often is coordinated by charged residues and hence the overall charge on the molecule is influenced by the neighboring side-chain groups which give the protein a varying net charge depending on the pH of the solute, which influences its binding and elution in an ion exchange column [22].

The pH and temperature profile of the enzyme demonstrates that the enzyme is pH and thermostable. Proteases with similar thermostability, pH activity, and stability enzymes have been reported by [23–25], where the enzyme retained 100–80% activity only for 30 min in temperatures from 35 to 60°C, while in our study the enzyme retained 100–70% activity after 1 h at 30–70°C demonstrating its unique thermostability at high temperatures.

The masses of the peptide fragments as obtained by MALDI TOF MS were typically constrained between 600 and 3000 Da relating to that the digested protein is of smaller molecular weight [8]. The amino acids modifications alter the residue mass (protein), possibly making the modified peptide more or less readily ionized, hydrophilic, or soluble. Sometimes, a chemical bond that is particularly labile to mass spectrometric fragmentation is introduced into the peptide sequence that makes the fragmentation of the peptide easier. However this results in increased number of false positives (incorrect identifications) and false negatives (missed true identifications), during identification of peptides. The error is minimized by employing Tandem mass spectrometry (MS/MS) that detects the fragmented ions in the spectra, thereby identifying the amino acid modification and peptide identification [26].

Significant *m*/*z* matchings were obtained in the database searching; however, some peptides exhibited no significant matching. High quality spectra may show unidentified peptides in a typical data analysis due to several reasons like smaller number of peptides generated due to improper digestion of protein, inaccurate charge state or precursor ion *m*/*z* measurement, constrained database search parameters, presence of chemical or posttranslational modifications, and incompleteness of the protein sequence database [27]. The presence of VVVHEMCHMV peptide in the most conserved region identifies the new enzyme as zinc metalloprotease. The majorities of zinc-dependent metallopeptidases share a sequence similarity and common pattern of primary structure in the zinc-binding region and can be grouped together as a superfamily known as the metzincins [3].

Secondary structure analysis by circular dichroism (CD) spectroscopy revealed presence of more *α* helices and random coils than *β* sheets. Protein CD spectra result in large part from peptide transitions associated with secondary structural components such as alpha (the alpha-helix is a 3.6_13_ helix), 3_10_ and 4.4_16_·3_10_ (other common helical conformations), polyproline-II helices, and parallel and antiparallel b-sheets. These produce characteristic CD spectral features in the ultraviolet (UV) wavelength regions through distinct interactions with the left and right circularly polarized light from a CD spectrophotometer or a synchrotron radiation circular dichroism (SRCD) beamline [28]. Because the spectra of these molecules in the far UV regions are dominated by the *n*-*π* and *π*-*π* ^*^ transitions of amide groups and are influenced by the geometries of the polypeptide backbones, their spectra are reflective of the different types of secondary structures present [29]. The alpha helix is the most stable of all the secondary structures, and this explains the extreme stable characteristic of the purified enzyme. *α*-helical structure has been reported to contribute in stability of enzyme produced by* Pseudomonas aeruginosa* strain *K* in organic solvents, broad range of temperatures, pHs, and metal ions [30]. Alpha helix proteases have been widely studied and reported to understand the diverse functionality of these enzymes [31, 32].

The results obtained from the CD spectroscopy were validated by the tertiary (3D) structure obtained from I-TASSER which also showed presence of more *α* helices and random coils than *β* strands. I-TASSER is an automated pipeline for protein tertiary structure prediction using multiple threading alignments and iterative structure assembly simulations [19].

The enzyme kinetics study revealed that the enzyme exhibited highest affinity towards casein, with a low *K*_*m*_ value suggesting that a minimum amount of enzyme was required for a maximum effect of reaction to occur. Low *K*_*m*_ values also suggest that the enzyme is normally saturated with substrate and will act at a constant rate, regardless of variations in the concentration of substrate within the physiological range [33]. Similar low *K*_*m*_ values have been reported by Gupta et al. [33] (*K*_*m*_ = 2.69 mg/mL, *V*_max⁡_ = 3.03 *μ*mol/min) and Tang et al. [25] (*K*_*m*_ = 3.97 mg/mL, *V*_max⁡_ = 7.58 *μ*mol/min) towards casein as substrate at 60°C indicating its high affinity and efficient catalytic role.

The salt tolerance of the enzyme shows that it is a mild halotolerant metalloprotease. Inouye et al. [34] have reported that NaCl enhances the catalytic activity and thermal stability of thermolysin. Halotolerant proteases have been reported by Shivanand and Jayaraman [35] and Ningthoujam and Kshetri [36]. Higher salinity may promote binding of a hydrophobic substrate to enzyme or of hydrophobic residues to each other within the enzyme to ensure optimal folding for enzymatic activity. Zinc metalloproteases with fibrinolytic and collagenolytic activity from various* Bacillus cereus* strains have been reported [24, 37].

The enzyme exhibited high stability in various organic solvents. Classical theory suggests that disulfide bonds stabilize proteins by reducing the entropy of the denatured state and hence they have been attributed for the unusual stability properties enzymes [38]. In the light of these findings, we presume that such disulfide bonds existing in the metalloprotease enzyme make it resistant against various organic solvents. Also the results show an enhanced enzyme activity in presence of some organic solvents. The general explanations offered include disruption of water structure in the vicinity of the active site formation of higher intrinsic activity resulting in as high as tenfold increase, or presence of high concentrations of miscible organic solvents may induce gross changes in substrate specificity and/or more subtle alterations in chiral selectivity [39, 40]. Metalloproteases showing stability in wide range of organic solvents have been reported [24, 31].

## 5. Conclusion

The enzyme studied in the present work was identified as a zinc metalloprotease as it exhibited 70–93% matching with zinc metalloprotease from various* Bacillus* sp. However, on the basis of its amino acid sequence, phylogenetic relationship and predicted tertiary structure, biochemical characteristics, and stability properties, it is established as a novel zinc metalloprotease.

The enzyme exhibited strong fibrinolytic and collagenolytic activity and thus finds a potential use as a natural agent for oral fibrinolytic therapy or thrombosis prevention, in the clinical therapy and in the wound healing process, and as experimental reagents in laboratory work. The enzyme was observed to be mild salt tolerant, while highly stable, in presence of organic solvents. Enzymes with such stability properties are very important from an industrial perspective. Halo tolerant proteases find application in food fermentation where NaCl is used as preservative. Salt stable collagenases containing ointment are frequently used in clinics to debris wounds. Nonaqueous enzymology has considerably enlarged the use of enzymes for a variety of applications. The unique solvent stability of the enzyme thus establishes it as promising biocatalyst for organic solvent-based enzymatic synthesis.

This enzyme can be exploited for a wide-ranged application in various industries owing to its stability, wide substrate specificity, and fibrinolytic and gelatinolytic properties.

## Figures and Tables

**Table 1:** Purification steps for metalloprotease.

	Activity (U)	Protein (mg)	Sp. activity U/mg	Recovery	Fold purification
Crude enzyme	31773.5	117.12	271.29	100	1
EtOH treated	31773.1	87.2	364.76	100	1.34
Sephadex	15682.7	10.4	1507.95	49.35	5.55
Q Sepharose	10668.9	3	3556.3	33.577	13.10

**Table 2:** The peptide matching (12/16 matches) for *m*/*z* data of metalloprotease with zinc metalloprotease from *Bacillus cereus* (gi∣507041200∣ref∣WP_016112858.1∣).

Spectrum	Prec.	MH+	Modifications	Missed cleavages	Position	Peptide
	*m*/*z*	Matched				
1.F2.2.1.5	2704.8679	2761.3123	MSO	0	1–23	MIHTYLGETINFHINCKKKKSVR (I)
1.F2.2.1.7	2383.300	2446.1576	CYS_CAM MSO	0	187–206	(R) VIDYVVVHEMCHMVHLNHDR (S)
1.F2.2.1.9	1638.686	1617.8601	MSO	1	39–51	(K) GTPVEYVLQLLEEK (W)
1.F2.2.1.15	1052.532	979.1244	CYS_CAM MSO	0	129–135	(R) FYYQQCK (A)
1.F2.2.1.12	1350.614	1314.5724		0	61–73	(K) EMKRVLGPQEK (V)
1.F2.2.1.13	1267.53	1260.4618	MSO	0	52–61	(K) WDWIQKTRK (E)
1.F2.2.1.16	868.4659	887.0877	MSO	0	136–143	(K) ALVEKSIK (S)
1.F2.2.1.3	1803.738	1786.1747	CYS_CAM MSO	0	172–186	(K) LQLTFNWKLAMAPPR (V)
1.F2.2.1.4	1707.594	1695.8900		0	24–38	(R) IYIDSYGNVEVQAPK (G)
1.F2.2.1.2	1765.5551	1740.1028		0	204–216	(R) SFWRLVGKIMPDYK (I)
1.F2.2.1.8	1838.717	1757.0606		0	110–123	(K) LHIYVKELEDEKIK (Q)
1.F2.2.1.1	1488.676	1494.6884	MSO	1	221–231	(K) EMEDWLALSSWK (M)

**Table 3:** Effects of organic solvents on the stability of the enzyme.

Solvents	log⁡*P* value	Day 1	Day 2	Day 4	Day 6	Day 8	Day 10
None		100	100	98.18	98	96	92
Hexane	3.98	99.26	81.37	96.71	51.29	40.21	38.98
Ethyl benzene	3.15	172.79	139.95	120.32	97.43	85.15	81.48
Xylene	3.15	106.37	182.84	176.40	160.28	132.17	118.39
Toluene	2.73	115.69	189.22	169.01	143.20	126.54	115.94
Benzene	2.13	99.95	95.58	96.95	87.22	86.54	83.1
Chloroform	2	107.6	107.84	84.457	25.94	24.52	18.93
Amyl alcohol	1.2	77.21	180.88	107.00	104.13	94.67	78.67
Butanol	0.88	94.12	154.41	125.35	104.13	14.04	8.41
Propanol 2	0.074	99.50	159.8	74.89	51.29	32.66	28.44
Acetone	−0.21	90.93	110.05	98.42	96.68	91.49	51.72
Ethanol	−0.235	85.29	100.24	88.62	61.53	50.07	38.98
Acetonitrile	−0.394	94.85	190.2	96.71	84.23	54.72	35.74
DMSO	−1.378	106.62	118.63	115.82	80.84	69.77	52.49
Glycerol	−1.93	147.30	95.83	93.77	85.11	76.54	21.58
